# Combination of mesenchymal stem cells and nicorandil: an emerging therapeutic challenge against COVID-19 infection-induced multiple organ dysfunction

**DOI:** 10.1186/s13287-021-02482-8

**Published:** 2021-07-15

**Authors:** Anahid Safari, Vicenzo Lionetti, Iman Razeghian-Jahromi

**Affiliations:** 1 Stem Cells Technology Research Center, Shiraz University of Medical Sciences, Shiraz, Iran; 2 Unit of Translational Critical Care Medicine, Institute of Life Sciences, Scuola Superiore Sant’Anna, Pisa, Italy; 3 UOS Anesthesiology and Intensive Care Medicine, Fondazione Toscana G. Monasterio, Pisa, Italy; 4 Cardiovascular Research Center, Shiraz University of Medical Sciences, Shiraz, Iran

**Keywords:** COVID-19, Mesenchymal stem cells, Nicorandil

## Abstract

The recent COronaVIrus Disease (COVID)-19 pandemic has placed an unprecedented burden on the drug development opportunity to prevent the onset of multi-organ failure.

Emerging experimental reports have highlighted the beneficial effects of mesenchymal stem cell (MSC) administration against COVID-19. MSCs and their derived exosomes may attenuate SARS-CoV-2-induced inflammatory response through managing the immune cell function and cytokine expression. Although these are promising results, the exposure of MSCs to chemical compounds with pharmacological activities may further improve their homing, survival, and paracrine machinery.

Nicorandil (N-[2-hydroxyethyl]-nicotinamide nitrate), an established adenosine triphosphate-sensitive potassium channel opener, is recently hypothesized to modulate inflammation as well as cell injury and death in COVID-19-affected lungs through inhibiting reactive oxygen species levels and apoptosis. Since it also exerts protective effects against hypoxia-induced MSC apoptosis, we assumed that transplanted MSCs combined to long-term nicorandil administration may survive longer in a severely inflamed microenvironment and have more beneficial effects in the treatment of SARS-CoV-2 infection than MSCs alone.

The recent COronaVIrus Disease (COVID)-19 pandemic has placed an unprecedented burden on the opportunity for improving therapy development to prevent the onset of multi-organ failure in SARS-CoV-2-infected patients.

Initial experimental reports revealed beneficial effects of mesenchymal stem cell (MSC) administration against COVID-19 [1], which attenuates severe acute respiratory coronavirus-2 (SARS-CoV-2) infection-induced inflammatory response through managing the immune cell function and cytokine profile [1]. Since MSCs are not infected by SARS-CoV-2 due to negative expression of angiotensin-converting enzyme 2, MSCs and their byproducts may prevent capillary barrier damage, depletion of the alveolar ATP levels, and bacterial growth in the inflamed pulmonary tissue [1]. Moreover, MSCs may enhance the repair of the fibrotic lung by means of a variety of secreted trophic factors [2]. However, the clinical efficacy of stem cell therapy is limited by poor engraftment and low survival rate of MSCs in the injured tissues. Therefore, different strategies have been tested to improve MSC survival and paracrine activity with conventional medications [3]. Although stem cells and drug combination therapy may also have synergistic effects in healing damaged lungs [2], selecting the right drug remains challenging.

Nicorandil (N-[2-hydroxyethyl]-nicotinamide nitrate), a nitric oxide (NO) donor and ATP-sensitive K^+^ channel opener with anti-inflammatory and anti-oxidant properties, is conventionally used to treat patients with ischemic heart diseases [4]. Recently, its use has also been hypothesized to prevent pulmonary inflammation as well as oxidation-induced cell injury in COVID-19 patients through inhibiting cytokine formation and apoptosis [5]. This suggestion is supported by preclinical reports demonstrating the protective role of nicorandil against acute lung injury via abolishing the activation of NF-κB and mitogen-activated protein kinase pathways in pulmonary artery endothelial cells [6]. Moreover, nicorandil can effectively increase the activation of phosphatidylinositol-3-kinase (PI3K)/Akt pathway, hypoxia-inducible factor, and superoxide dismutase up to heal damaged pulmonary tissue in rats [7].

Although mechanisms underlying COVID-19 are still under investigation, regulatory effects of the abovementioned cellular signaling may impact on the pathogenesis, severity, and long-term sequelae of severe SARS-CoV-2-induced pneumonia [8].

In light of its pleiotropic protective effects [4, 6, 7], we can assume that nicorandil may be a safe drug to combine with MSC transplantation in treating COVID-19 patients with multiple organ dysfunctions.

Interestingly, nicorandil also promotes MSC survival under conditions mimicking the ischemic microenvironment, such as hypoxia and oxidative stress, through the activation of the PI3K/Akt signaling pathway and the reduction of reactive oxygen species production [9]. Moreover, the combination of nicorandil and MSCs displays greater protective effects in a rodent model of heart failure compared with stem cells alone [10]. Therefore, it is conceivable that nicorandil administration in severe COVID-19 patients treated with MSC transplantation may enhance their beneficial effects on vital organs.

Lastly, we cannot exclude that paracrine factors may play a key role in mediating synergistic effects of combined MSC-nicorandil therapy. In particular, exosomes, the smallest extracellular vesicles that serve as mediators for cell-to-cell communication, recapitulate the efficacy of MSCs in treating critical illness [11]. The first pilot study revealed that exosomes derived from allogenic bone marrow MSCs downregulate cytokine storm and reverse hypoxia in severe COVID-19 patients [12]. Considering the broad potential values of MSCs and nicorandil, future randomized controlled trials are mandatory to validate the possibility for a promising combinational therapeutic approach that might counteract the multiple organ dysfunction syndrome in COVID-19 patients via paracrine mechanisms.

## Data Availability

Not applicable

## Abbreviations

COVID: COronaVIrus Disease
MSCs: Mesenchymal stem cells
NO: Nitric oxide
PI3K: Phosphatidylinositol 3 kinase
SARS-CoV-2: Severe acute respiratory syndrome coronavirus-2

## References

[1] Liu S, Peng D, Qiu H, Yang K, Fu Z, Zou L (2020). Mesenchymal stem cells as a potential therapy for COVID-19. Stem Cell Res Ther..

[2] Chuang HM, Ho LI, Harn HJ, Liu CA (2021). Recent findings on cell-based therapies for COVID19-related pulmonary fibrosis. Cell Transplant..

[3] Cantoni S, Cavallini C, Bianchi F, Bonavita F, Vaccari V, Olivi E (2012). Rosuvastatin elicits KDR-dependent vasculogenic response of human placental stem cells through PI3K/AKT pathway. Pharmacol Res..

[4] Horinaka S (2011). Use of nicorandil in cardiovascular disease and its optimization. Drugs..

[5] Ashour H, Elsayed MH, Elmorsy S, Harb IA (2020). Hypothesis: the potential therapeutic role of nicorandil in COVID-19. Clin Exp Pharmacol Physiol..

[6] He M, Shi W, Yu M, Li X, Xu J, Zhu J (2019). Nicorandil attenuates LPS-induced acute lung injury by pulmonary endothelial cell protection via NF-κB and MAPK pathways. Oxid Med Cell Longev..

[7] Kseibati MO, Shehatou GSG, Sharawy MH, Eladl AE, Salem HA (2020). Nicorandil ameliorates bleomycin-induced pulmonary fibrosis in rats through modulating eNOS, iNOS, TXNIP and HIF-1alpha levels. Life Sci..

[8] Chan CM, Ma CW, Chan WY, Chan HY (2007). The SARS-coronavirus membrane protein induces apoptosis through modulating the Akt survival pathway. Arch Biochem Biophys..

[9] Zhang F, Cui J, Lv B, Yu B (2015). Nicorandil protects mesenchymal stem cells against hypoxia and serum deprivation-induced apoptosis. Int J Mol Med..

[10] Mohamed SS, Ahmed LA, Attia WA, Khattab MM (2015). Nicorandil enhances the efficacy of mesenchymal stem cell therapy in isoproterenol-induced heart failure in rats. Biochem Pharmacol.

[11] Terrasini N, Lionetti V (2017). Exosomes in critical illness. Crit Care Med..

[12] Sengupta V, Sengupta S, Lazo A, Woods P, Nolan A, Bremer N (2020). Exosomes derived from bone marrow mesenchymal stem cells as treatment for severe COVID-19. Stem Cells Dev..

